# Comparison of two label-free global quantitation methods, APEX and 2D gel electrophoresis, applied to the *Shigella dysenteriae *proteome

**DOI:** 10.1186/1477-5956-7-22

**Published:** 2009-06-29

**Authors:** Srilatha Kuntumalla, John C Braisted, Shih-Ting Huang, Prashanth P Parmar, David J Clark, Hamid Alami, Quanshun Zhang, Arthur Donohue-Rolfe, Saul Tzipori, Robert D Fleischmann, Scott N Peterson, Rembert Pieper

**Affiliations:** 1 Pathogen Functional Genomics Resource Center, J Craig Venter Institute, 9704 Medical Center Drive, Rockville, MD 20850, USA; 2 Cummings School of Veterinary Medicine, Tufts University, 200 Westboro Road, North Grafton, MA 01536, USA

## Abstract

The *in vitro *stationary phase proteome of the human pathogen *Shigella dysenteriae *serotype 1 (SD1) was quantitatively analyzed in Coomassie Blue G250 (CBB)-stained 2D gels. More than four hundred and fifty proteins, of which 271 were associated with distinct gel spots, were identified. In parallel, we employed 2D-LC-MS/MS followed by the label-free computationally modified spectral counting method APEX for absolute protein expression measurements. Of the 4502 genome-predicted SD1 proteins, 1148 proteins were identified with a false positive discovery rate of 5% and quantitated using 2D-LC-MS/MS and APEX. The dynamic range of the APEX method was approximately one order of magnitude higher than that of CBB-stained spot intensity quantitation. A squared Pearson correlation analysis revealed a reasonably good correlation (*R*^*2 *^= 0.67) for protein quantities surveyed by both methods. The correlation was decreased for protein subsets with specific physicochemical properties, such as low M_r _values and high hydropathy scores. Stoichiometric ratios of subunits of protein complexes characterized in *E. coli *were compared with APEX quantitative ratios of orthologous SD1 protein complexes. A high correlation was observed for subunits of soluble cellular protein complexes in several cases, demonstrating versatile applications of the APEX method in quantitative proteomics.

## Introduction

Until recently, quantitative proteomics studies have mainly relied on two-dimensional (2D) gel electrophoresis combined with protein identification by mass spectrometry (MS) to analyze large datasets of proteins from complex protein mixtures [1,2]. Quantitation of relative protein abundances from 2D gels has involved the comparison of protein spot intensities across two or more sample groups [3]. Limited dynamic range caused by low detection sensitivity, the saturation of protein staining, and insufficient spot resolution from overlapping and co-migrating protein spots have confounded the accuracy and depth of protein quantitation in 2D gels [4,5]. In addition, proteins with certain physicochemical traits are difficult to analyze in 2D gels, including those with a basic pI value, a high or low M_r _value, and transmembrane domains. Alternative protein quantitation strategies based on shotgun proteomics have evolved to address some of these limitations [6,7], including peptide or protein labeling [8,9], and label-free strategies [10].

Label-free approaches have included measurements of mass spectral peak intensities [11] and spectral counting [12]. While peak intensities of peptide ions can be correlated with protein abundances, spectral counting methods estimate protein abundances by comparing the number of MS/MS spectra assigned to each protein, based on the assumption that the number of peptides observed from a protein correlates with its abundance [13]. Spectral counting provides the advantage of measuring both relative [10] and absolute abundances of different proteins in complex samples [14]. To account for the fact that larger proteins contribute more peptides compared to smaller proteins, spectral counting data is normalized to avoid abundance over-estimation of high M_r _proteins [13,15]. However, since the ionization efficiency of peptides and their subsequent observation in the mass spectrometer depend on a variety of factors including their physicochemical properties, peptide composition and local chemical environment [9], spectral counting based solely on the number of experimentally observed, proteotypic peptides is often not an accurate measure of protein abundance [16,17].

To address this, the APEX methodology, a label-free quantitation method for absolute protein expression measurements was developed by the Marcotte group [14,18]. The APEX quantitation method correlates spectral counts obtained from mass spectrometric data with computational predictions of proteotypic peptides for each protein to estimate protein abundance from the fraction of observed peptide mass spectra. For proteotypic peptide prediction, machine learning classification algorithms are applied to a training dataset comprised of peptides from a limited set of abundant proteins to build a classification model for the prediction of proteotypic peptides generated *in silico *from the entire proteome. Prior expectation of observing these peptides and the confidence in protein identification serve as correction factors in APEX quantitation. APEX thereby estimates absolute protein concentration as the proportionality between the abundance of a protein and the number of its proteotypic peptides versus that of the total protein concentration and all proteotyic peptides [14].

In this study, we quantitatively analyzed the proteome of the Gram-negative bacterium *Shigella dysenteriae *serotype 1 (SD1) using two different approaches: (1) 2D gel display and quantitation of proteins *via *spot intensities; (2) tryptic digestion of the proteome, and LC-MS/MS in conjunction with APEX to estimate protein abundances from quantitation of peptides. The human pathogen SD1 is the most virulent of the four *Shigella *species and a causative agent of shigellosis [19,20]. The predicted number of proteotypic peptides for each SD1 protein was derived from a species-specific SD1 training dataset generated from 100 abundant SD1 proteins, employing a recently developed software application based on the APEX methodology termed the APEX Quantitative Proteomics Tool [21]. The APEX tool is freely available, user-friendly and easily downloadable for quantitation of proteins using LC-MS/MS datasets. We also describe a method to estimate protein abundances derived from CBB-stained 2D spot intensity values as molecules per cell. These experiments enabled us to generate a comparative proteomic dataset from two label-free global quantitation methods. Furthermore, we observed a high correlation of known stoichiometric ratios of subunits for several characterized *E. coli *protein complexes and the APEX ratios of equivalent SD1 proteins. These findings are significant as they demonstrate that computationally modified spectral counting methods, such as APEX, are among the most promising developments in quantitative proteomics.

## Materials and methods

### Bacterial strains and culture conditions

The strain Sd1617 of *Shigella dysenteriae *serotype 1 (SD1) was grown to stationary phase in Luria-Bertani (LB) medium at 37°C and pelleted by centrifugation at 7,000 × *g *for 10 min at 4°C. The SD1 cell pellet was washed with PBS by centrifuging at 6,000 × *g *for 15 min at 4°C and resuspended in a hypotonic lysis buffer composed of 25 mM Tris-HCl, pH 7.8 with 150 μg/mL lysozyme, 0.05% Triton X-100, 5 mM EDTA and protease inhibitors benzamidine (1 mM) and AEBSF (1 mM). After incubation in the lysis buffer for 30 min at room temperature (RT), the samples were immediately stored at -80°C until further processing. For nucleic acid digestion, bacterial samples suspended in the lysis buffer were thawed and gently agitated for 1 h at RT after the addition of leupeptin, DNAse and RNAse (10 μg/mL each) and 20 mM MgCl_2_. Cell lysates were centrifuged at 16,000 × *g *for 30 min at 4°C, and the supernatant containing bacterial cell lysate proteins was recovered.

### 2D-LC-MS/MS analysis of SD1 cell lysate

Following cell lysis, the extracted bacterial proteins were precipitated in six volumes of ice-cold acetone at -20°C for at least 1 h. Acetone-precipitated proteins were recovered as a pellet after centrifugation at 5,000 × *g *for 10 min. The protein pellet was resuspended in 0.1 M TAB (triethyl ammonium bicarbonate, Sigma Chemicals, St. Louis, MI) buffer, pH 8.5, and the protein concentration determined using the BCA assay (Sigma Chemicals). Proteins were denatured in 0.1% SDS and reduced using 5 mM TCEP (Tris(2-carboxyethyl)phosphine) for 1 h at 37°C, followed by alkylation using 10 mM MMTS (methyl methanethiosulfonate) for 1 h at RT [22]. In-solution trypsin digestion of the complex protein mixture was performed by the addition of trypsin at 1:25 for 5 h at 37°C followed by 1:50 digestion overnight. Peptide digests (*ca*. 100 μg) were fractionated by 2D-LC-MS/MS, first on an offline Polysulfoethyl-A SCX column (4.6 × 50 mm, Nest Group, USA). Fractions collected from the SCX separation were then delivered from 96-well plates to a RP-C_18 _column (BioBasic C_18_, 75 μm × 10 cm, New Objective, USA), online with an ion trap mass spectrometer (LTQ, ThermoElectron). Spectra were acquired in automated MS/MS mode with the top five parent ions selected for fragmentation. LC-MS/MS was performed in three sequential *m/z *subscans (300–650, 650–900, 900–1500 *m/z*) to increase the sampling depth [14]. MS/MS data from sequential runs were combined for analysis and searched by the Mascot search engine (Matrix Science) against a *S. dysenteriae *Sd197 database, a subset created from a non-redundant NCBI protein database. Mascot search parameters allowed for tryptic specificity of up to one missed cleavage, with methylthio-modifications of cysteine as a fixed modification and oxidation of methionine as a variable modification. Mascot search results of three replicate 2D-LC-MS/MS experiments were validated by PeptideProphet™ and ProteinProphet™ [23] which are part of the Trans-Proteomic Pipeline (TPP) accessed at http://tools.proteomecenter.org/wiki/index.php?title=Software:TPP.

### Quantitation of a ten protein mixture using the APEX method

A ten protein standard mixture was initially used to assess the accuracy of the computational quantitation performed with the APEX Quantitative Proteomics Tool [21]. Proteins were mixed in known concentrations ranging from 1 to 500 pmol in 0.1 M TAB, pH 8.5, denatured in 0.1% SDS, reduced with 5 mM TCEP for 1 h at 37°C, alkylated with 10 mM MMTS for 1 h at RT, and digested with trypsin (1:50) at 37°C overnight. The resulting peptides were analyzed by LC-MS/MS (LTQ) in three sequential *m/z *subscans (300–650, 650–900, 900–1500 *m/z*). LC-MS/MS data from three replicate runs were searched by Mascot against a NCBInr database, and the Mascot results validated by PeptideProphet™ and ProteinProphet™ analyses [23]. Employing the APEX tool [21], a training dataset was generated, *O*_*i *_values calculated, and APEX abundances estimated by normalizing for the measured total protein concentration, as described in more detail for the APEX quantitation of SD1 proteins.

### APEX quantitation from LC-MS/MS data of SD1 cell lysates

The APEX quantitation of SD1 proteins using the APEX Quantitative Proteomics Tool consisted of three steps: building a SD1 training dataset, computing SD1 protein *O*_*i *_(expected number of unique proteotypic peptides for protein *i*) values, and calculating SD1 protein APEX abundances. Proteins in the training dataset were chosen based on the 100 most abundant SD1 proteins in order to generate a species-specific training dataset. A list of the top 100 SD1 proteins was generated based on high spectral counts per protein and high protein and peptide identification probabilities [18]. The training dataset .ARFF file was constructed based on 35 peptide sequence attributes including mass, length, pI, charge, hydrophobicity measures, amino acid composition, amino acid frequencies within secondary peptide structures and other peptide physicochemical properties deemed significant for the computational prediction of proteotypic peptides [14,17]. The list of all 35 peptide physicochemical attributes is provided to users of the APEX tool at http://pfgrc.jcvi.org/index.php/bioinformatics/apex.html.

To compute SD1 protein *O*_*i *_values, the Random Forest classifier algorithm available from the Weka data mining software package at http://www.cs.waikato.ac.nz/ml/weka was employed. Random Forest is the default classifier algorithm of the APEX tool due to its high performance [14]. The classifier algorithm was applied to the SD1 training dataset constructed in the previous step, and then to all tryptic peptides generated *in silico *from the SD1 proteome to enable computation of SD1 protein *O*_*i *_values. APEX abundances of the SD1 proteins observed by 2D-LC-MS/MSwere calculated using the protXML file generated from the PeptideProphet™ and ProteinProphet™ validation of the Mascot search results and the SD1 protein *O*_*i *_values. A <5% false positive rate (FPR) was chosen, along with a normalization factor of 2.5 × 10^6^. The normalization factor in the APEX tool is equivalent to the term *C *in the APEX equation [14], which represents the total concentration of protein molecules per cell. Since *S. dysenteriae *is very closely related to *E. coli*, the total number of protein molecules/cell estimated at 2–3 × 10^6 ^for *E. coli *[14] was used as a normalization factor in the APEX abundance measurements of *S. dysenteriae *proteins.

### 2D gel analysis of SD1 cell lysate

Following cell lysis, the extracted SD1 proteins were analyzed in 2D gels and by MS as described previously [24,25]. Briefly, *ca*. 110 μg of protein was loaded onto 24 cm IPG strips (GE Healthcare) with pI range 4–7. The first-dimension protein separation in IPG strips and the second-dimension (SDS-PAGE) polyacrylamide slab gel separation (25 × 19 × 0.15 cm), as well as the Coomassie Brilliant Blue G-250 (CBB) gel staining and scanning procedures, were performed as described previously [24,25]. For protein spot detection, scanned 2D gel images were analyzed by the gel image analysis software Proteomweaver v.4.0 (Bio-Rad). Tryptic peptides extracted from protein gel plugs of interest were analyzed by MALDI-TOF/TOF (4700 Proteomics Analyzer, Applied Biosystems), as well as LC-MS/MS (LTQ, ThermoElectron) interfaced with a nano-LC system (Agilent). The Mascot search engine was employed to search data against the *S. dysenteriae *Sd197 database, and the results viewed in an in-house LIMS system. MS protein identifications were matched to the excised protein spots. The 2D spots that matched to a single protein with high confidence were considered for quantitative comparison with APEX estimations of protein abundances.

### Estimation of protein abundances from 2D gel spot intensities

Automatic detection, quantitation and determination of CV (coefficient of variation) for protein spots (n = 3) were performed using the software Proteomweaver v.4.0. The methods were previously described in detail [24]. Spot matching was confirmed by extensive analysis of all spots by MS. We modified the software-based methodology for relative quantitation of proteins from 2D spot intensities [26] to estimate absolute abundances of SD1 proteins as molecules per cell. Data from other studies on *E. coli*, *Yersinia pestis *and SD1 (unpublished data) revealed that approximately 75% of the total proteome of γ-proteobacteria is visualized in pH range 4–7 2D gels. Thus, we estimated the protein abundance of any protein *i *(2DE_*i*_) from 2D spot intensity values as follows:

where the numerator I_*i *_is the (average) spot intensity of any protein *i*, while the denominator represents the total spot intensity of all spots detected. As in the APEX calculations, the term *C *represents the total number of protein molecules per cell (estimated to be 2.5 × 10^6^) or the measured total protein concentration in the sample [14]. This approach allowed us to convert relative spot intensity volumes into protein abundances (molecules/cell) that were used for the comparative quantitative analysis with the APEX method.

## Results

### Comparison of APEX-computed protein quantities with known quantities of a ten protein standard mixture

A ten protein mixture consisting of bovine α-casein (10 pmol), bovine cytochrome *c *(20 pmol), bovine serum albumin (40 pmol), bovine deoxyribonuclease (500 pmol), chicken lysozyme (5 pmol), chicken ovalbumin (100 pmol), equine myoglobin (60 pmol), rabbit glycogen phosphorylase (2 pmol), human transferrin (1 pmol) and human carbonic anhydrase I (200 pmol) was digested and analyzed by LC-MS/MS. The average number of MS/MS spectra was 10218 from three replicate analyses. APEX-calculated protein abundance estimates correlated well with the injected protein concentrations, with Spearman rank correlation coefficient *R*_*s *_= 0.98 and squared Pearson correlation coefficient *R*^*2 *^= 0.92 (Additional File 1). Interestingly, the APEX values for proteins in the low molarity range (1–20 pmol) were more precise than those for proteins with high molarities (500 pmol), possibly attributable to the saturation of MS/MS spectral sampling at very high protein concentrations. The correlations dropped significantly (*R*_*s *_= 0.79, *R*^*2 *^= 0.68) when APEX abundances were estimated without the calculation of *O*_*i *_values (*O*_*i *_= 1), emphasizing the importance of accurate *O*_*i *_(expected number of unique proteotypic peptides for protein *i*) values for reliable protein abundance measurements.

### Profile of SD1 proteins in Coomassie-Blue-stained 2D gels and quantitative analysis

**Figure 1:**
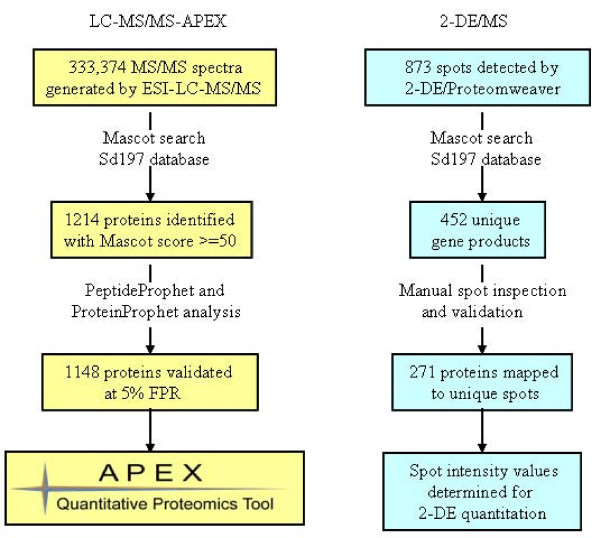
**Flow chart of data analysis approach**. The data analysis approaches employed for the LC-MS/MS-APEX and 2-DE/MS methodologies are shown here. The numbers in both approaches represent the average of three replicate experiments. 1148 SD1 proteins were quantitated by LC-MS/MS-APEX and 271 proteins from 2D gels.

**Figure 2:**
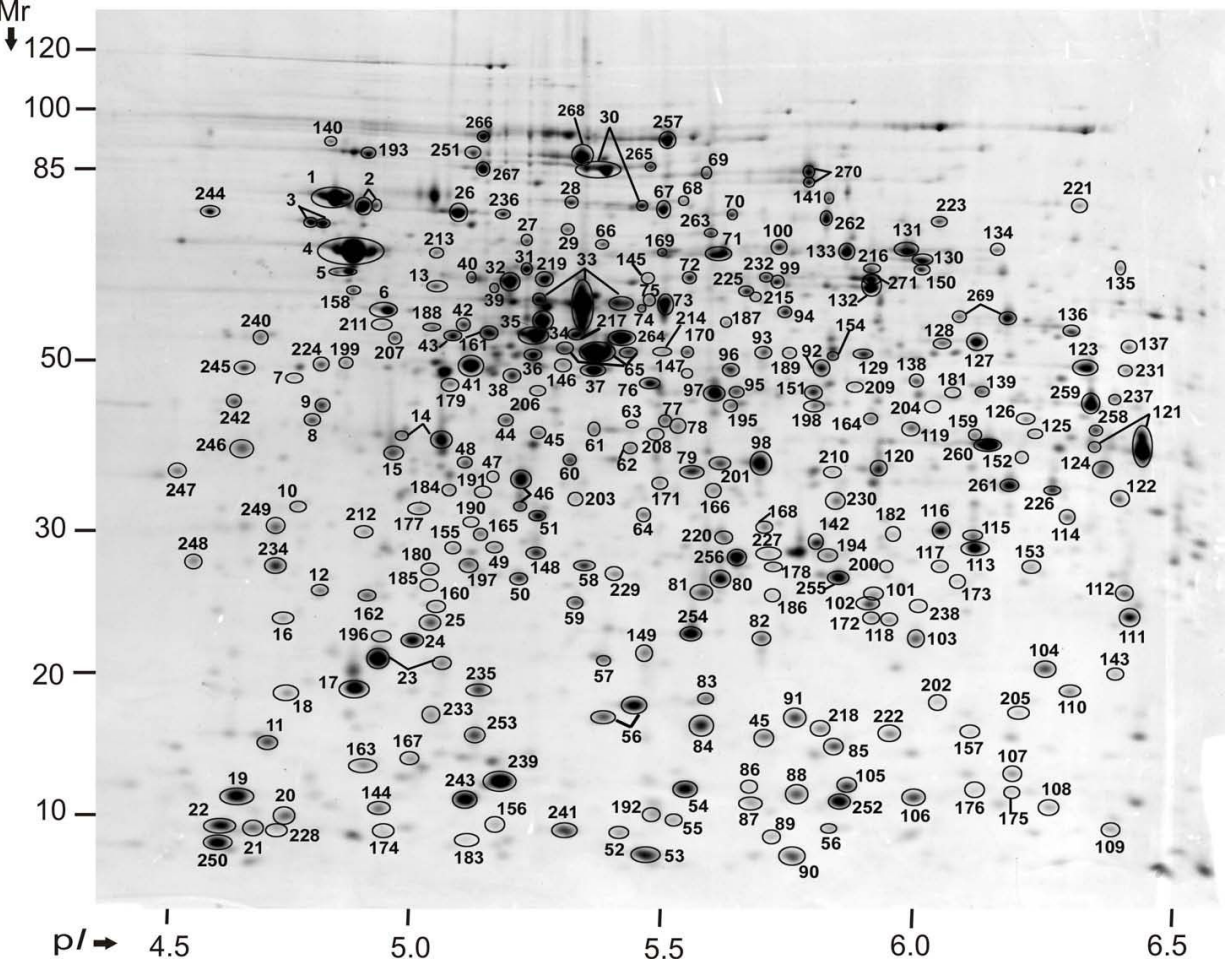
**Annotated proteome map of 2D gel**. SD1 proteins were analyzed by 2-DE in the pH range 4 – 7. 271 proteins mapped to the gel by 2-DE/MS are represented by spot numbers. 2-DE protein abundances of 255 proteins matched to unique spots were correlated with APEX abundances.

Nearly 880 spot features were matched among all three 2D gels subjected to protein spot-based quantitative analysis. Most of these features, identified *via *LC-MS/MS and MALDI-TOF/TOF-MS, collapsed into 452 unique gene products (Fig. 1), primarily because a significant number of proteins were assigned to multiple 2D gel spots. These variants represented products of protein degradation and amino acid side chain modification events [2]. In addition, the resolution capacity of 2D gels in a pH range from 4 to 7 and a M_r _range from 200 to 6 kDa is limited, resulting in co-migration of proteins in specific 2D gel areas. Since protein modifications and spot overlaps confound 2D gel-based protein abundance analysis, we thoroughly checked all spot identifications and the level of protein co-migration in gels. As a result of this validation step, only 271 proteins assigned to one protein species per spot were selected for quantitative assessments in 2D gels. These proteins are denoted in Fig. 2 with numbers equivalent to those provided in the 2D gel annotation table (Additional File 2). Highly abundant proteins frequently matched to more than two spots, e.g. GroEL, DnaK, GadB, OsmY and TufA (spots numbered 4, 30, 33, 56 and 65, Fig. 2). In such cases, the sum of spot intensities contributed to the overall protein abundance estimates.

For a correlation analysis with the APEX method, relative abundances of CBB-stained 2D gel spots and spot trains were converted to molecule/cell estimates. An equation described in the Materials and Methods section was used for this conversion, based on an estimate of 2.5 × 10^6 ^total protein molecules per cell and on the simplifying assumption that individual proteins were stained with CBB with roughly equal efficiency. From these calculations, the most abundant proteins in 2D gels were GroEL, GadB and TufA, each with >35,000 molecules/cell. These proteins are indeed known to be highly abundant in stationary phase cells of γ-proteobacteria [27,28]. Surveyed as the least abundant proteins were the putative sugar-dephosphorylating enzyme YidA (gene locus SDY_4179) and the galactose-binding transport protein MglB, with <550 molecules/cell (Additional File 2).

### Profile of SD1 proteins using 2D-LC-MS/MS and APEX for quantitative analysis

333,374 MS/MS spectra (average of three datasets) were generated by the 2D-LC-MS/MS analysis of SD1 proteins. Among the 1214 proteins identified from Mascot searches of LC-MS/MS runs, 1148 proteins were validated by the algorithms PeptideProphet™ and ProteinProphet™, assuming a FPR of <5%. Thirty-five of these proteins were derived from the virulence-associated pSD1 plasmid, including invasion plasmid antigens and type III secretion system components. More than 250 hypothetical proteins were identified demonstrating that the corresponding genes were indeed expressed. The coverage of the genome-predicted SD1 proteome was *ca*. 26%. This dataset was subjected to protein quantitation using the APEX Quantitative Proteomics Tool (Fig. 1). The Random Forest classifier algorithm was trained on a high quality training dataset of 100 abundant proteins to predict protein *O*_*i *_values. The algorithm classified *ca*. 23% of the peptides in the training dataset as 'observed', compared to *ca*. 9% reported previously [18]. In addition, the 'observed' peptides were predicted with a *F*-measure of 0.75 (0.72 precision and 0.8 recall), while 'non-observed' peptides were predicted with a much higher *F*-measure (0.94 precision and 0.91 recall). This increased the overall accuracy of correct classifications on the training dataset by the classifier to *ca*. 88%. These results supported the notion that the proteins chosen for the training dataset resulted in the identification of a large number of proteotypic peptides, which in turn permitted better estimation of protein abundances.

APEX abundance values were calculated using SD1 protein-specific *O*_*i *_values normalized by an estimated total number of 2.5 × 10^6 ^protein molecules/cell [14]. The proteins are listed in the APEX protein quantitation table (Additional File 3). The most abundant proteins were the DNA-binding protein HU-alpha (HupA), the global regulator Dps and the PTS system protein PtsH, each estimated at >30,000 molecules (*ca*. 1.2% of total protein/cell). GroEL, GadB and TufA, the most abundant proteins from 2D gel measurements, also yielded high copy numbers (*ca*. 25,000 molecules, 1% of total protein/cell) using the APEX method. Estimates for the 100 least abundant proteins were in the range of 20 to 250 molecules per cell (*ca*. 0.001% to 0.01% of total protein/cell). For example, formate acetyltransferase 3 (TdcE), the Fe-S subunit of a putative oxidoreductase (YffG), and the large subunit of glutamate synthase (GltB) were calculated to be present at less than 30 molecules/cell. The dynamic range of APEX-based protein abundance measurements was 10^3^, about one order of magnitude higher than that of CBB-stained spot intensity quantitation from 2D gels. Correlation of SD1 protein APEX estimates with protein properties such as isoelectric point (pI) and net charge followed previously reported trends, with no significant correlation observed for these protein properties [14]. Apparently, the combination of LC-MS/MS and APEX introduces little bias in abundance measurements based on protein characteristics such as protein pI or net charge. Of note, the APEX *vs*. 2D gel comparison of proteins with pI values >7 is of limited value, because most proteins are not displayed in the pH range of gels examined here (4 to 7).

### Biological and biochemical implications of APEX protein abundance data

SD1 protein abundances estimated by the APEX tool correlated inversely with protein M_r _values, as seen for GltB (M_r _= 163,330) with a APEX_*GltB *_estimate of less than 30 molecules/cell and HupA (M_r _= 9535) with a APEX_*HupA *_estimate of more than 40,000 molecules/cell. This trend was previously reported [14]. It has been speculated that smaller proteins are present in higher copy numbers in cells than larger proteins, as a way to minimize transcriptional and translational costs [29]. To assess APEX abundance measurements in the context of subunit stoichiometries for characterized multi-subunit protein complexes, we compared protein abundance ratios from the SD1 APEX dataset with stoichiometric ratios designated for orthologous *E. coli *protein complexes. Unless noted otherwise, the *E. coli *protein complex data were derived from the EcoCyc database [30] at http://www.ecocyc.org. As shown in Table 1, most stoichiometric ratios of soluble subunits of intracellular protein complexes determined by APEX deviated less than 20% from the reported stoichiometric ratios. This data supported the precision of APEX-based protein quantitation for soluble proteins such as chaperones (e.g. HslU/HslV [31]), polymerase complex subunits (e.g. RpoA/RpoB [32]) and subunits of protein assemblies involved in energy metabolism (e.g. AtpA/AtpD and SucC/SucD [33]).

**Table 1:** Stoichiometric ratios of protein complexes as quantitated by APEX

^(*a*) ^Protein complex	^(*b*)^*E. coli *stoichiometric ratio	SD1 APEX ratio	^(*c*) ^SD1 APEX abundances (± *sd*) (molecules/cell)
SucC/SucD	1:1	1:1.01	6889(± 827):7004(± 651)

AccD/AccA	1:1	1:1.03	2012(± 302):2086(± 121)

AccC/AccB	1:2	1:2.12	1534(± 549):3258(± 460)

AceF/AceE	1:1	1:1.04	6716(± 739):7029(± 707)

SdhB/SdhA	1:1	1:1.21	4869(± 740):5917(± 44)

HslU/HslV	1:2	1:1.98	1486(± 125):2953(± 955)

RpoB/RpoA	1:2	1:2.05	2095(± 301):4312(± 1016)

AtpD/AtpA	1:1	1:1.01	8713(± 216):8848(± 673)

AtpH/AtpG	1:1	1:1.41	1052(± 295):1491(± 595)

NlpB/YaeT	1:1	1:1.46	477(± 25):699(± 76)

AhpF/AhpC	1:1 or 1:5	1:6.02	2272(± 216):13673(± 303)

YaeT/NlpB/SmpA/YfiO/YfgL	1:1:1:1:1	2.8:1.9:4.6:1:10.6	699:477:1157:250:2655

AtpA/AtpD/AtpG/AtpH	3:3:1:1	8.4:8.3:1.4:1	8848:8713:1491:1052

*Shigella dysenteriae *(SD1) subunit ratios of protein complexes ^(*a*) ^quantitated by APEX were compared with previously reported stoichiometric ratios for orthologous *E. coli *protein complexes ^(*b*)^. APEX abundances were derived from three datasets, with standard devations (*sd*) included ^(*c*)^.

For a few protein complexes, the observed APEX stoichiometry was different from the reported ratio. The thioredoxin peroxidase AhpC/AhpF is composed of an equimolar dimer-dimer assembly according to the EcoCyc database, but the observed APEX ratio was 6:1. Interestingly, further review of the literature suggested decamer formation of AhpC in a reduced state, whereas the dimer is formed in an oxidized state [34]. Thus, the examined stationary phase growth state of SD1 cells appeared to favor the reduced, active AhpC state, which is linked to reduction of hydroperoxide substrates. Correlation decreased for ratios of subunits that formed part of membrane-associated protein complexes. The integral outer membrane protein YaeT and four lipoproteins (NlpB, SmpA, YfiO and YfgL) each supposedly contribute a monomer to a five-protein outer membrane complex. The APEX quantitated stoichiometry of proteins in this complex was 2.8:1.9:4.6:1:10.6, respectively. A similar case was seen for subunits of the F_1_-ATP synthase complex. In comparison to AtpA and AtpD, the subunits AtpG and AtpH revealed lower APEX-calculated quantities than those expected from the reported stoichiometry of 3:3:1:1 (AtpA:AtpD:AtpG:AtpH) [35], with the observed stoichiometry being 8.4:8.3:1.4:1. Presumably, the causes were differences in the efficiency of extracting individual subunits from membranes during cell lysate preparation, with AtpA and AtpD being more soluble peripheral membrane proteins [36]. Stoichiometric ratios of subunits of four protein complexes were also determined from 2D gel data. They deviated more from the expected ratios than those determined by the APEX method. For example, the stoichiometric ratios for SucC/SucD and SdhB/SdhA were 1:1.42 and 1:1.53 (2D gel), and 1:1.01 and 1:1.21 (APEX), respectively, whereas the expected ratios are 1:1 for both protein complexes.

### Comparison of protein profiles and quantitative data derived from APEX and 2D gel analyses

Quantitative data using LC-MS/MS and APEX were obtained for 1148 proteins, 4.2-fold greater than that for 2D gels. Ninety-four percent of all proteins quantitatively assessed in 2D gels were also part of the APEX dataset. In each of the SD1 datasets, most of the proteins were soluble, cytoplasmic or periplasmic, according to PSORTb [37] predictions. In cases where the PSORTb analysis was inconclusive, the datasets were queried with four other algorithms (TMHMM [38], SignalP [39], LipoP [40] and BOMP [41]) to predict subcellular protein localizations. Approximately 82% and 93% of the proteins in the APEX and 2D gel datasets, respectively, were predicted to be soluble. Approximately 18% and 7% of the proteins in the APEX and 2D gel datasets, respectively, were predicted to be membrane-integrated. Of note, we observed and quantitated ten times as many membrane-integrated SD1 proteins by APEX compared to the 2D gel method (212 *vs*. 20 proteins). The squared Pearson correlation coefficient for the quantitation of soluble proteins *vs*. all proteins did not vary significantly (*R*^*2 *^= 0.66 *vs*. 0.67). The correlation for membrane proteins was not performed, since only 15 membrane proteins were shared between the APEX and 2D gel datasets. For all 255 proteins with APEX and 2D gel data points, absolute abundance estimates are listed in the comparative analysis table (Additional File 4). In summary, the APEX data revealed fewer constraints than 2D gel data regarding the ability to quantitate proteins with physicochemical characteristics very different from mean values, particularly for proteins with high hydropathy scores, high net charges, high pI and low M_r _values (Table 2). The difficulties to quantitate such proteins in 2D gels are extensively documented [42].

**Table 2:** Comparison of proteins quantitated by 2D-LC-MS/MS-APEX *vs*. 2-DE

	2D-LC-MS/MS-APEX	2-DE
Cytoplasmic	883	234

Periplasmic	52	17

Cytoplasmic membrane	132	14

Outer membrane	80	6

Extracellular	1	-

Plasmid proteins	35	5

Hypothetical proteins	257	35

Abundance range (molecules/cell)	~20 to ~45000	~500 to ~52000

M_r _range (kDa)	6.4 – 163.3	8.3 – 99.7

pI range	3.59 – 11.81	4.52 – 8.48

Net charge range	33.74 to -50	3.65 to -40

Hydropathy range	1.36 to -1.53	0.31 to -1.53

Aromaticity range	0.01 to 0.18	0.01 to 0.14

1148 SD1 proteins quantitated by 2D-LC-MS/MS-APEX were compared with 271 proteins quantitated in 2D gels based on physicochemical properties and protein subcellular localizations. APEX data revealed less constraints than 2D gel data in quantitation of proteins with physicochemical characteristics very different from mean values, including high hydrophobicity, very low M_r_, high net charge and high pI values.

### Protein physicochemical properties affect APEX *vs*. 2D gel abundance correlations

**Figure 3:**
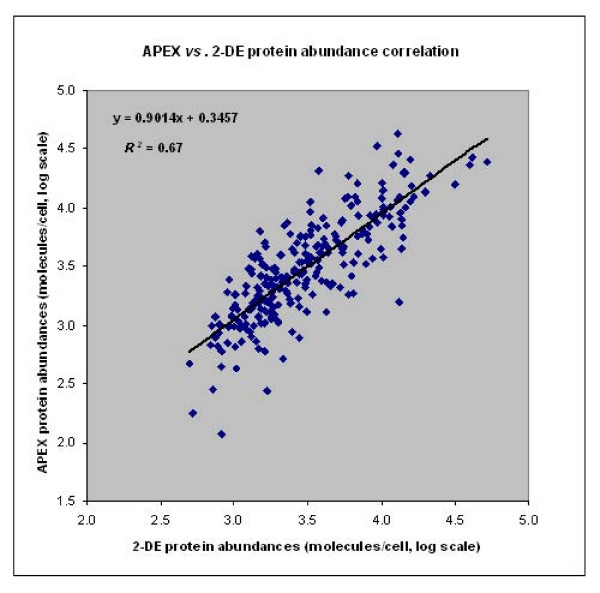
**Correlation of protein abundances estimated by APEX *vs*. 2-DE**. 255 SD1 proteins common to both the APEX and 2-DE datasets were correlated for protein abundance estimations by the two methodologies for an overall Spearman rank correlation of *R*_*s *_= 0.81 and squared Pearson correlation of *R*^*2 *^= 0.67.

The distribution of log scale protein abundance plotted as a function of various protein physicochemical properties (M_r_, hydropathy, aromaticity, etc.) followed a similar pattern overall for the APEX and 2D gel datasets. The correlation for all of the 255 shared proteins comparing 2D gel and APEX datasets revealed relatively good correlation values (*R*_*s *_= 0.81, *R*^*2 *^= 0.67) (Fig. 3). For quantitative assessments on low abundance proteins (<1000 molecules/cell by APEX), the correlation decreased dramatically (*R*_*s *_= 0.17, *R*^*2 *^= 0.08, n = 31). Interestingly, the correlation for high abundance proteins such as GroEL, GadB, TufA (>10,000 molecules/cell) was also poor (*R*_*s *_= 0.29, *R*^*2 *^= 0.13, n = 33). Decreased correlation for lower abundance proteins was not unexpected, given poor staining sensitivity in 2D gels, which coupled with low spectral counts and a limitation to one or two unique proteotypic peptides (APEX) also compromised quantitation accuracy [4]. Decreased correlation for high abundance proteins may have resulted from variability in the saturation of 2D spot staining and saturation of proteotypic peptide detection *via *LC-MS/MS-APEX. In support of the latter hypothesis, the protein with the highest molarity (500 pmol) included in the 10-protein standard mixture was also quantitatively underestimated *via *APEX.

**Figure 4:**
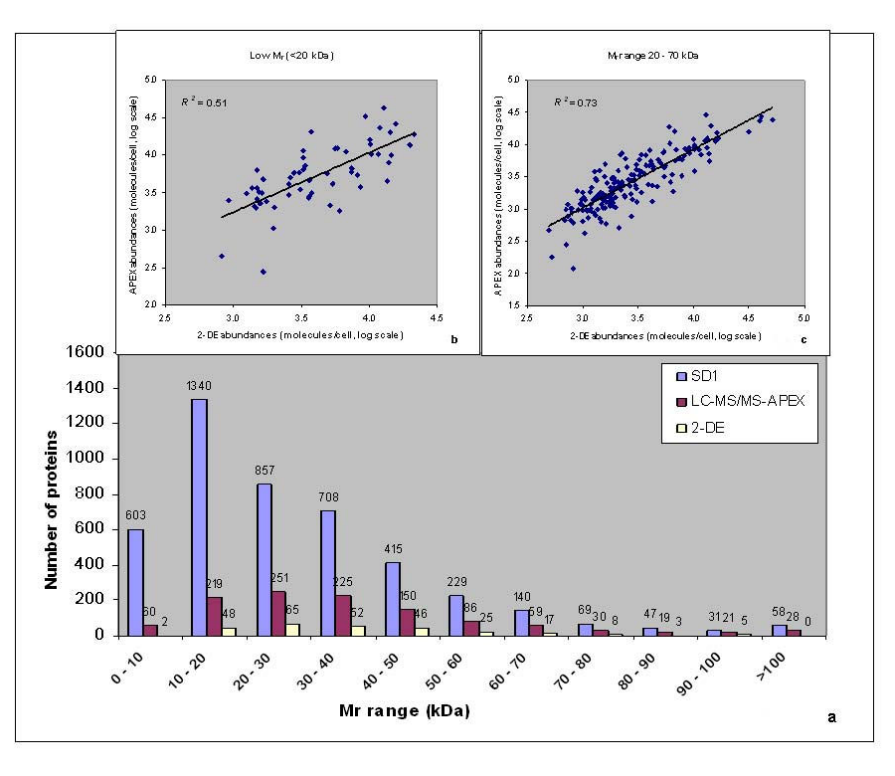
**Correlation of protein abundances estimated by APEX *vs*. 2-DE based on protein M_r_**. Proteins quantitated by APEX and 2-DE were compared against all proteins predicted for the Sd197 genome sorted by M_r _in bins of 10 kDa width (Fig. 4a). Correlation of protein abundances estimated by APEX *vs*. 2-DE decreased for low M_r _proteins at <20 kDa (*R*^*2 *^= 0.51), but increased for proteins in the 20 – 70 kDa M_r _range (*R*^*2 *^= 0.73) compared to the overall abundance correlation (*R*^*2 *^= 0.67).

To determine the bias of each method towards quantitating proteins with specific physicochemical properties [43], we correlated data for distinct ranges of protein M_r _values, hydropathy, aromaticity, pI and net charges. For M_r _values <10 kDa and >100 kDa, only two proteins were reliably quantitated in 2D gels, compared to 88 proteins quantitated *via *APEX (a 1:44 ratio) (Fig. 4a). The numerical ratios (2D gels *vs*. APEX) ranged from 1:3 to 1:7 for proteins binned in 10 kDa intervals for the 10 to 100 kDa range. As shown in Fig. 4b, the correlation of protein abundances in the <20 kDa range was markedly lower (*R*_*s *_= 0.71, *R*^*2 *^= 0.51) than that of protein abundances in the 20–70 kDa range (*R*_*s *_= 0.85; *R*^*2 *^= 0.73). Criteria such as ineffective protein precipitation with acetone during sample preparation for tryptic digests/LC-MS/MS and ineffective fixation and/or staining of small proteins in 2D gels appeared to impact correlation scores for small proteins. The fact that small proteins highly abundant in the stationary growth phase of *E. coli*, such as PtsH [44] and Dps [45], yielded *ca*. three-fold higher molecule/cell values *via *APEX compared to 2D gels, suggested a quantitation inaccuracy of 2D gel spots in the M_r _region below 20 kDa. The correlation was also decreased for high M_r _proteins (>70 kDa, *R*_*s *_= 0.65, *R*^*2 *^= 0.38). In the context of 2D gels, proteins with M_r _values >90 kDa occasionally fail to migrate into the 2^nd ^dimension gel and also form extensive spot trains due to pI- and M_r_-altering modifications (e.g. ClpB and PflB). Such events result in the underestimation of protein quantities [46,47].

**Figure 5:**
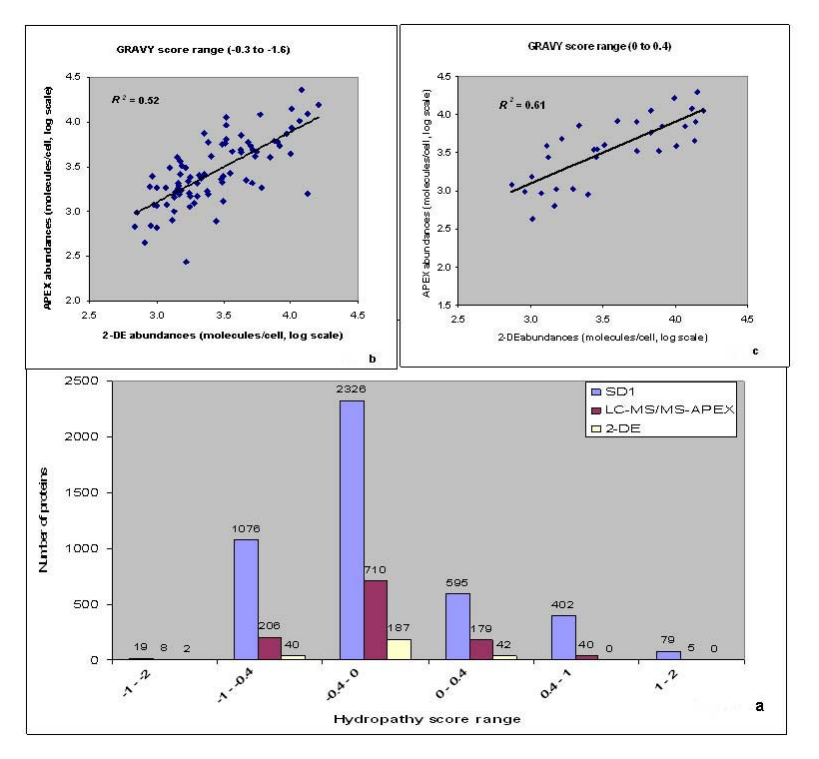
**Correlation of protein abundances estimated by APEX *vs*. 2-DE based on hydropathy score**. Hydropathy (GRAVY) score of a protein was calculated as the arithmetic mean of the sum of the hydropathic indices of each amino acid. The left end of the scale in Fig. 5a represents hydrophilic proteins, and the right end of the scale represents hydrophobic proteins. Within a particular hydropathy range, the number of proteins quantitated by APEX and 2-DE were compared against all protens predicted for Sd197. Correlation of protein abundances estimated by APEX *vs*. 2-DE decreased for very hydrophobic (*R*^*2 *^= 0.61) and also for very hydrophilic proteins (*R*^*2 *^= 0.52) compared to the overall abundance correlation.

**Figure 6:**
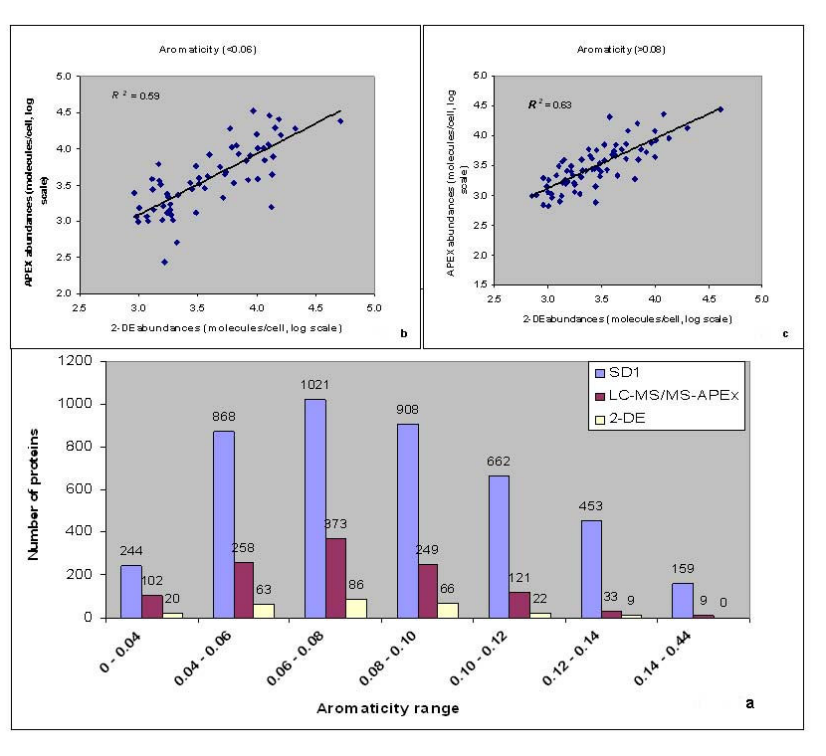
**Correlation of protein abundances estimated by APEX *vs*. 2-DE based on aromaticity**. Proteins predicted for Sd197 genome were sorted based on the frequency of aromatic amino acids F, W and Y in proteins and compared with proteins quantitated by APEX and 2-DE (Fig. 6a). Correlation of protein abundances estimated by APEX *vs*. 2-DE decreased with increase in non-polarity (>0.08 aromaticity values, *R*^*2 *^= 0.63) and increase in polarity (<0.06 aromaticity values, *R*^*2 *^= 0.59) compared to the overall abundance correlation.

Hydrophobic transmembrane domain and lipoproteins are difficult to analyze in 2D gels due to their limited solubility in isoelectric focusing (IEF) experiments. This includes the precipitation of proteins with GRAVY (grand average of hydropathy) scores >0.4 during IEF [48]. Incomplete solubilization and digestion of hydrophobic proteins also compromise their quantitation *via *2D-LC-MS/MS and APEX, as pointed out in the section on protein complexes. The GRAVY score was used to sort proteins based on their hydrophobicity or hydrophilicity [49]. According to predictions of GRAVY scores for the Sd197 proteome, 76% of all proteins were observed to be hydrophilic (GRAVY score <0). The comparison of APEX *vs*. 2D gel data indicated advantages of the APEX method for the quantitation of hydrophobic proteins. Forty-five proteins with GRAVY scores >0.4 were quantitated with APEX, in contrast to zero proteins in 2D gels in this score range (Fig. 5a). An improved correlation of protein abundances compared to the overall correlation was observed for 142 moderately hydrophilic proteins with a GRAVY score range of 0 to -0.3 (*R*_*s *_= 0.85, *R*^*2 *^= 0.73). The correlation decreased not only with increasing hydrophobicity of proteins, such as Pfs and YhbL (GRAVY scores >0, *R*_*s *_= 0.77, *R*^*2 *^= 0.61, Fig. 5c), but also with increasing hydrophilicity (GRAVY score <-0.3, *R*_*s *_= 0.72, *R*^*2 *^= 0.52, Fig. 5b). The cause of the decreased correlation in the context of hydrophilic proteins was unclear. To determine the effect of protein aromaticity [50] on abundance correlation, proteins were sorted based on the frequency of aromatic amino acids F, W and Y in proteins http://codonw.sourceforge.net/index.html. Proteins predicted in the Sd197 genome ranged in their aromaticity values from 0.01 to 0.43 (Fig. 6a). Correlation of protein abundances improved moderately for proteins with aromaticity values of 0.06 to 0.08 (*R*_*s *_= 0.85, *R*^*2 *^= 0.71) in comparison to that for all proteins. Most of the proteins in this aromaticity range were also hydrophilic (GRAVY score >0). Correlation decreased with increasing non-polarity (aromaticity values >0.08, *R*_*s *_= 0.78, *R*^*2 *^= 0.63, Fig. 6c) and increasing polarity (e.g., RpoZ and OsmY, aromaticity values <0.06, *R*_*s *_= 0.76, *R*^*2 *^= 0.59, Fig. 6b). Since protein hydropathy and protein aromaticity are related properties, abundance correlations based on aromaticity and hydropathy values followed a similar trend.

In contrast to 2D gels, proteins identified by 2D-LC-MS/MS included the alkaline pI range (Table 1). Our ability to compare proteins quantitated in 2D gels *vs*. APEX was compromised by the fact that proteins in 2D gels were only focused in the pI range of 4–7, thus excluding basic proteins from a meaningful quantitative analysis. The distribution of proteins detected by the APEX method followed a bimodal pattern with two distinct clusters for acidic proteins *vs*. basic proteins. Proteins with pI values in the pH range 7–8 are relatively rare due to their lower solubility at a near-neutral net charge under physiological growth conditions. Most of the predicted proteins for the Sd197 genome were observed in the 5–6 pI range, as predicted for other organisms [43], and reflected in the relative distribution of proteins quantitated by the APEX method and in 2D gels. The *R*_*s *_and *R*^*2 *^values for distinct pI ranges of proteins with pI values <7 did not deviate from the correlation for all proteins. Net charge of a protein at pH 7 was then calculated to determine the correlation of protein abundances based on charge. About 96% of the Sd197 proteins were predicted in the net charge range of -20 to 20 units, with *ca*. 94% of the proteins quantitated by APEX and in 2D gels within that range. Proteins with a net positive charge >20 and <-40 (at pH 7) were particularly rare in the 2D gel dataset. The *R*_*s *_and *R*^*2 *^values for net charge ranges were in good agreement with those observed for distinct pI ranges. The correlation of APEX *vs*. 2D gel abundance measurements for moderately acidic proteins in the net charge range from 0 to -10 (*R*_*s *_= 0.82, *R*^*2 *^= 0.66, n = 164), and for strongly acidic proteins (*R*_*s *_= 0.78, *R*^*2 *^= 0.67, n = 84) was close to that of the overall correlation, indicating no quantitative bias based on protein pI or net charge.

In summary, the evaluation of qualitative and quantitative data comparing APEX and 2D gels revealed several advantages of the APEX method: (1) higher detection sensitivity of the digested peptides *via *LC-MS/MS compared to proteins in CBB-stained 2D gels; (2) fewer constraints in the detection of peptides featuring a variety of physicochemical characteristics per protein (APEX) compared to that of proteins *via *2D gel spots; (3) higher dynamic range of peptide spectral counts (LC-MS/MS) than that of proteins detected in CBB-stained 2D gel spots. Other computationally adjusted LC-MS/MS spectral counting methods [51,52] may perform as well as APEX for global protein quantitation. Although these methods also employ peptide detectability, they were explored only in the context of relative quantitation, rather than absolute quantitation as performed by the APEX method. With appropriate adjustments to sample preparation procedures, shortcomings of the APEX method regarding quantitation of hydrophobic and membrane-bound proteins can likely be addressed. On the other hand, unlike LC-MS/MS-based methods, 2D gels retain the advantage that post-translational modification processes and functional characteristics of proteins are often measurable qualities and useful in interpreting biological processes.

## Discussion

While 2D gels have been used for more than 40 years for highly parallel protein quantitation, the APEX method was developed very recently by integrating spectral counting with computational predictions of proteotypic peptides from LC-MS/MS datasets to estimate protein abundances [14]. In this report, proteomic datasets derived from cell lysates of *S. dysenteriae *serotype 1 were subjected to a direct comparison of these label-free quantitation methods. Applying the APEX Quantitative Proteomics Tool [21] to a high quality training dataset of 100 high abundance SD1 proteins ensured that optimal parameters and *Oi *values were established for the SD1 APEX quantitation. In-depth analysis of MS data obtained from replicate 2D gels also served as a quality control step. Proteins whose spot assignments were not reproducible or revealed evidence for extensive spot overlaps were not included in the APEX *vs*. 2D gel correlation analysis.

Strategies to enable absolute quantitation of proteins from 2D gels have involved radioactive labeling of proteins and scintillation counting of protein spots [53], while fluorescent dyes have been generally employed for relative protein quantitation (differential display), e.g. 2D-DIGE [46]. Previous studies comparing APEX with 2D gel abundance measurements from 2D-DIGE and radioactive labeling resulted in lower correlations of *R*^*2 *^= 0.21 for 210 *E. coli *proteins and *R*^*2 *^= 0.52 for 48 yeast proteins [14]. The usual quantitative analysis mode of CBB-stained 2D gels is also differential display which results in spot quantitation relative to another dataset. In this study, a direct label-free comparison of abundance measurements (APEX *vs*. CBB-stained 2D gels) was performed, which required the estimation of absolute protein abundances derived from relative spot quantities in 2D gels. This was achieved *via *an equation incorporating a factor estimating total protein molecules/cell corrected by the estimated ratio of gel-visualized *vs*. total protein per sample. A nonlinear relationship between spot intensity volumes and actual protein amounts has been mentioned as a caveat for measurements of accurate protein abundance in 2D gels [3,43]. This pertains to the fact that spot staining saturation occurs for highly abundant proteins and to the notion that individual proteins differ in their affinity to the staining dye used. The dataset on highly abundant SD1 proteins resulted in a decreased correlation with APEX values, compared to the correlation for the entire SD1 dataset, suggesting that saturation effects may have compromised the accuracy of CBB-stained 2D spot intensity measurements. More sensitive fluorescent dyes such as SYPRO Ruby increase the dynamic range of protein abundance measurements in 2D gels and reduce the problem of spot saturation. In theory, this could result in improved protein abundance correlations with the APEX method. Technical problems, however, often limit the value of using a more sensitive 2D gel dye. Such problems include insufficient spot resolution, which is detrimental to the quantitation of low abundance proteins, and the requirement of high resolution imaging systems to detect the increased dynamic range of fluoresecent dye-stained 2D spots. CBB is still a widely used dye for 2D gel-based proteomic studies [54,55] and, therefore, a good first choice for the APEX *vs*. 2D gel-based comparative analysis.

The overall correlation between APEX- and 2D gel-based protein abundances yielded a *R*_*s *_value of 0.81 and a *R*^*2 *^value of 0.67. In comparison to the correlation for all 255 proteins, abundance correlations increased for subsets of proteins with distinct physicochemical properties. Based on protein M_r _values, correlation of abundance estimates improved for 182 proteins in the M_r _range 20 – 70 kDa (*R*^*2 *^= 0.73), while the correlation decreased considerably for low M_r _proteins (*R*^*2 *^= 0.51). Very low M_r _(<15 kDa) and very high M_r _(>100 kDa) proteins are more challenging to quantitate, for reasons better known in the context of 2D gels [1], such as inefficient fixing and staining of low M_r _proteins, and modifications of amino acid residues giving rise to multiple variants of high M_r _proteins. During sample preparation for 2D-LC-MS/MS, protein loss due to ineffective acetone precipitation of low M_r _proteins may result in the underestimation of protein quantities. Of note, protein abundances estimated by APEX correlated inversely with protein M_r _[14]. The underlying reasons appear to be biological rather than technical [29]. Schmidt *et al*. [43] reported that 2D gel analysis and ICAT-LC/MS, a peptide-based quantitation relying on isotope-labeled cysteine residues in proteins, each resulted in underestimation of proteins with M_r _values <10 kDa. Our data support the notion that, if a low M_r _protein has several unique proteotypic peptides with high identification probabilities by LC-MS/MS, the APEX method is well suited for quantitation (e.g. YjbJ with a M_r _= 8.3 kDa in this dataset). In contrast, a low M_r _protein with a small number of proteotypic peptides (e.g. EmrR with a M_r _= 20.5 kDa in this dataset) may be less accurately measured by the APEX method.

Limitations in the quantitation of alkaline and hydrophobic proteins in 2D gels have been described previously [1]. Due to the fact that the examined pI range of 2D gels was 4 – 7 in this study, the correlation analysis was more applicable to hydrophobic proteins than to basic proteins. The correlation between APEX and 2D gel datasets decreased with high protein hydrophobicity. There is considerable evidence for wide-spread quantitative underestimation of hydrophobic proteins in 2D gels [1]. Such proteins are usually membrane-integrated or membrane-anchored, characteristics that lower protein solubilization and resolution in 2D gels. In the 2D gel dataset, 7.3% of the identified proteins were predicted to be membrane-associated, while the membrane-associated proteins formed 18.5% of the APEX dataset. Also, for very hydrophobic proteins such as Pfs and YhlB (hydropathy score >0.3) quantitated in the common protein dataset, abundance estimates in 2D gels were *ca*. two- to threefold lower than the equivalent APEX abundance measurements. This is in contrast to a report by Schmidt *et al*. [43] where 2D gels overestimated proteins in the hydrophobic range compared to ICAT-LC/MS. Inadvertent mislabeling of hydrophobic and hydrophilic score ranges in a figure pertaining to this experiment, however, may be the explanation (Jungblut, personal communication). Interestingly, the comparison of stoichiometric ratios of protein subunits that were part of soluble and membrane protein complexes allowed us to assess 2D-LC-MS/MS-APEX measurement accuracies. The stoichiometric ratios for the examined membrane protein complexes deviated more from the expected values than the ratios for soluble protein complexes. Likely causes of the differences in ratios comparing APEX values *vs*. known stoichiometric ratios of *E. coli *membrane protein complexes were ineffective protein solubilization and/or tryptic digestion. We cannot exclude the possibility that hydrophobic peptide analysis by LC-MS/MS followed by APEX computational adjustments also influenced the measurement accuracy of membrane protein complexes. Quantitative subunit ratios were unavailable for all but four protein complexes in the 2D gel dataset and deviated more from the expected ratios compared to the corresponding APEX dataset.

We are not aware of other reports comparing LC-MS/MS-based, computationally modified protein quantitation data with quantitation from CBB-stained 2D gel spot intensity data. Our study demonstrates a generally good correlation between 2D gel and APEX quantitative measurements. The combination of APEX and 2D gels in proteomic analyses is of interest because these methodologies are inexpensive, versatile and bypass chemical or isotope-labeling steps that can introduce more experimental variability in quantitative analysis experiments. The combination of quantatitive 2D gel and APEX analyses is a powerful tool in proteomics research. 2D gels provide the advantages of visual proteome representation and easy detection of protein isoforms with modifications resulting from M_r _and pI changes, which are often biologically significant [1,56]. Examples observed here are: (1) the periplasmic protein Agp (spot # 35, Fig. 2) whose spot pI precisely matches that of a protein N-terminally truncated by 22 amino acids, indicative of signal peptide cleavage; (2) the chaperone/protease ClpB (spot # 30, Fig. 2), which was displayed in isoforms, one with an N-terminal truncation of *ca*. 160 amino acids; this N-terminal region has been linked to a binding site critical for activation of ClpB [57]. The APEX method, which is more sensitive and has a higher dynamic range of quantitation, yields comprehensive protein abundance data. APEX also shows promise for determination of stoichiometric ratios of subunits part of protein complexes. We demonstrated that the ratios of subunits of a variety of soluble protein complexes derived from APEX measurements were close to the experimentally reported stoichiometries. We also discussed an example where the stoichiometric ratio of a protein complex, the peroxidase AhpC/AhpF, implied a specific structure-function relationship. The observed 6:1 APEX ratio (AhpC:AhpF) suggested a reduced, active state of AhpC associated with substrate reduction [34]. In proteomics, such quantitative data is ideally combined with parallel analysis of native protein complexes, e.g. BN-PAGE [58], a tool that directly reveals participation of proteins in a specific complex. However, BN-PAGE is not as sensitive and quantitatively accurate as the APEX method. In conclusion, we identified an additional area in protein research where APEX will be a useful discovery tool.

## Abbreviations

2-DE: two-dimensional electrophoresis; APEX: Absolute protein expression; ARFF: Attribute relation file format; CBB: Coomassie Brilliant Blue; FPR: False positive rate; IEF: Isoelectric focusing; LIMS: Laboratory Information Management System; LC-MS/MS: Liquid chromatography with tandem mass spectrometry; MALDI-TOF/TOF: Matrix-assisted laser desorption ionization with tandem time of flight; MS: Mass spectrometry; MMTS: Methyl methanethiosulfonate; M_r_: protein molecular weight; *O*_*i*_: estimation of expected number of unique proteotypic peptides for a given protein *i*; pI: protein isoelectric point; *R*^*2*^: squared Pearson correlation coefficient; *R*_*s*_: Spearman rank correlation coefficient; RT: Room temperature; SD1: *Shigella dysenteriae *type 1; TAB: Triethyl ammonium bicarbonate; TCEP: (Tris(2-carboxyethyl)phosphine).

## Competing interests

The authors declare that they have no competing interests.

## Authors' contributions

SK – project conception and implementation, researched APEX technique, sample prep, generation of 2D-LC-MS/MS datasets and quantitation using the APEX Quantitative Proteomics Tool, spot quantitation of 2-DE gels, bioinformatic analysis of LC-MS/MS-APEX and 2-DE datasets, comparison of APEX and 2-DE quantitations, primary manuscript author, JCB – software engineering and development of the APEX Quantitative Proteomics Tool, computational analysis of APEX and 2-DE datasets, manuscript review, PPP – sample prep, performed 2-DE of samples, SH – analysis of 2-DE gels, DJC – LC-MS/MS analysis of samples, HA – MALDI analysis of 2-DE samples, QZ – provided bacterial samples, manuscript review, AD – provided bacterial samples, manuscript review, ST – provided bacterial samples, manuscript review, RDF – project oversight, manuscript review, SNP – project oversight, manuscript review, RP – project conception and implementation, participation in data interpretation and writing of the manuscript.

## Supplementary Material

Additional file 1 **Additional Table S1**. Correlation of APEX estimates of a ten protein standard mixture with known protein concentrations ranging from 1 – 500 pmol (*R*_*s *_= 0.98, *R*^*2 *^= 0.92). Click here for file

Additional file 2 **Additional Table S2**. Protein abundance estimates from 2-DE spot intensity quantitation. 271 proteins are listed with 2D gel spot numbers, accession numbers, gene names, locus tags, protein description, abundance values, physicochemical properties and subcellular localizations. 16 proteins unique to the 2D gel analysis are in bold letters. Click here for file

Additional file 3 **Additional Table S3**. Protein abundance estimates from APEX quantitation. 1148 proteins are listed with the false positive error rate, *p*_*i*_, *n*_*i*_, *O*_*i *_and APEX abundance values calculated using the APEX Quantitative Proteomics Tool. The corresponding gene names, locus tags, physicochemical properties and subcellular localizations are also listed in the table. Click here for file

Additional file 4 **Additional Table S4**. Comparison of SD1 protein abundance estimates from APEX *vs*. 2-DE quantitations for 255 common proteins listed along with 2D gel spot numbers, gene names, locus tags, physicochemical properties and subcellular localizations. Click here for file
